# Supplementary material for: On-surface synthesis of poly(p-phenylene ethynylene) molecular wires via in situ formation of carbon-carbon triple bond
Source: Nat Commun. 2018 Jun 13;9:2322. doi: 10.1038/s41467-018-04681-z (PMC5998079; doi:10.1038/s41467-018-04681-z)
Supplement: Supplementary file 1 — Supplementary Information [file 41467_2018_4681_MOESM1_ESM.pdf]

Supplementary Information for

On-surface synthesis of poly(*p*-phenylene ethynylene) molecular wires *via in situ* formation of carbon-carbon triple bond

Shu *et al.*

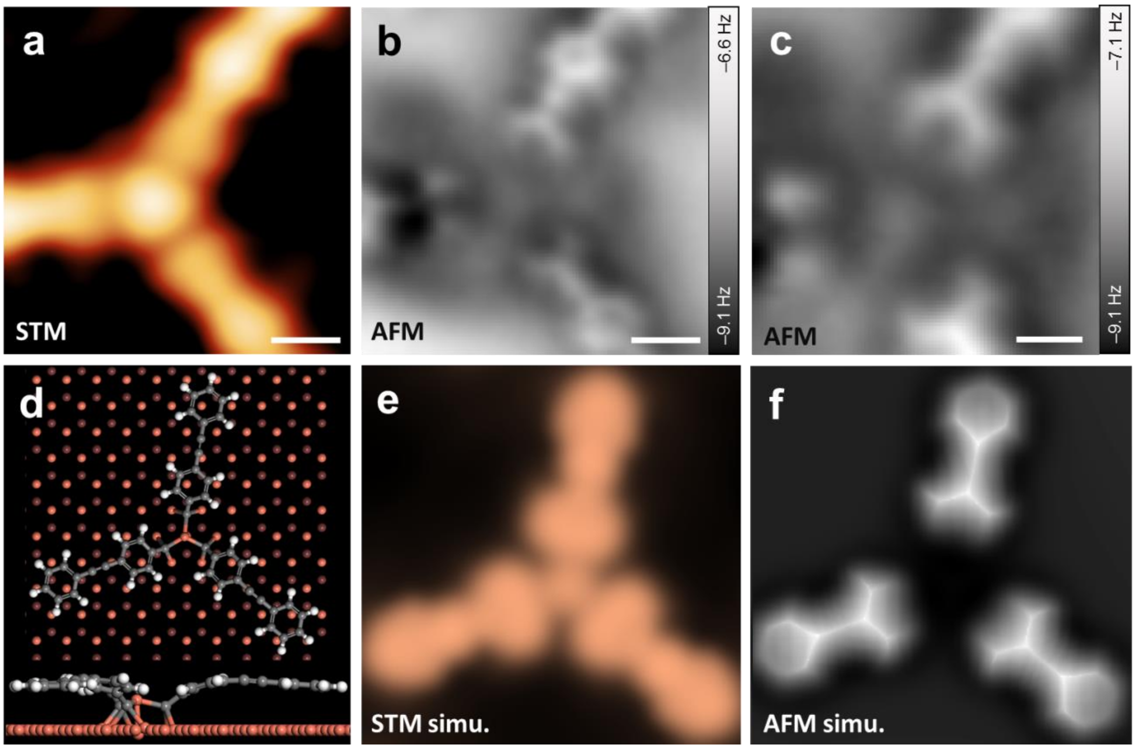

Supplementary Figure 1 | The bonding configuration of branched structure observed in Figure1b. (a) STM image of branched structure showing a bright protrusion in the joint point of three-fold nodes. (b, c) The corresponding nc-AFM images acquired with CO-functionalized tip illustrating the adjacent phenylenes tilted downward close to substrate and the joint point appeared featureless. According to the earlier publication[1], the protrusion is deduced to be a Cu adatom due to the bright contrast in STM image, and the branched structure could be assigned to a surface-supported coordination structure consisting of three PPE nanowires and a three-coordinate Cu center. (d) Top- and side-view DFT-optimized model of the three-fold branched structure. (e, f) STM and AFM simulations based on the optimized structure, agreeing well with the experimental results. The centered Cu adatom and the carbon atoms directly bonding to Cu adatoms which were lower than the PPE nanowires on Cu(111) cannot be clearly resolved in nc-AFM image. It is noteworthy that the optimized Cu adatom located at the top site of Cu(111). The termini of PPE bound to Cu center is carbyne radical and needs to be bonded with three neighboring Cu atoms, thus the Cu adatom located on the top site is the most stable structure in this three-fold coordination node. Scale bars: a, 500 pm; b, 500 pm; c, 300 pm.

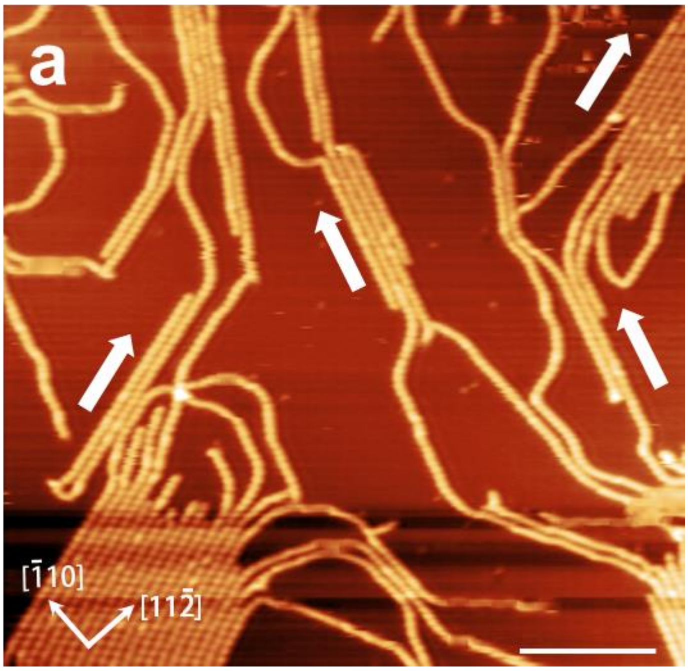

Supplementary Figure 2 | Large-scale STM images of PPE nanowire arrays after annealing at 358 K. (a) The raft-like PPE nanowire arrays were distributed with mixed orientation along the preferred directions (the orientations deviating by $\pm 19°$ from $[0\bar{1}1]$ or the equivalent orientations of Cu(111)). Scale bar: 10 nm.

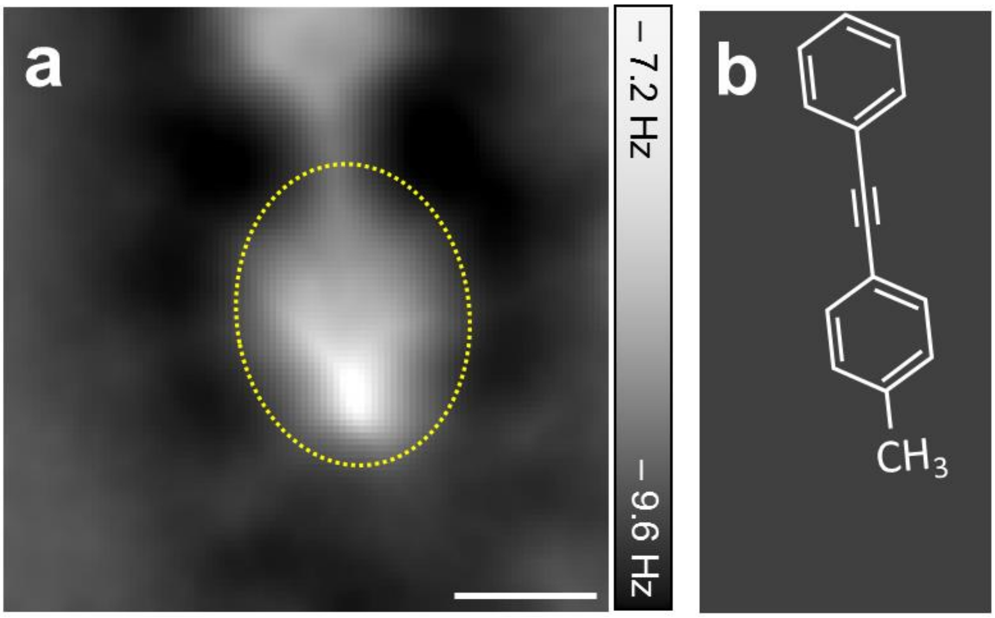

Supplementary Figure 3 | The recognition of PPE nanowire terminus. (a) Nc-AFM image of PPE nanowire terminus. Scale bar: 400 pm. (b) The corresponding atomic model. After annealing at 358 K, the functional group linked to terminal phenylene in PPE nanowire shows bright feature in nc-AFM image. According to earlier publication[2], the functional group could be identified as -CH_3, which was formed by being passivated by hydrogen atoms.

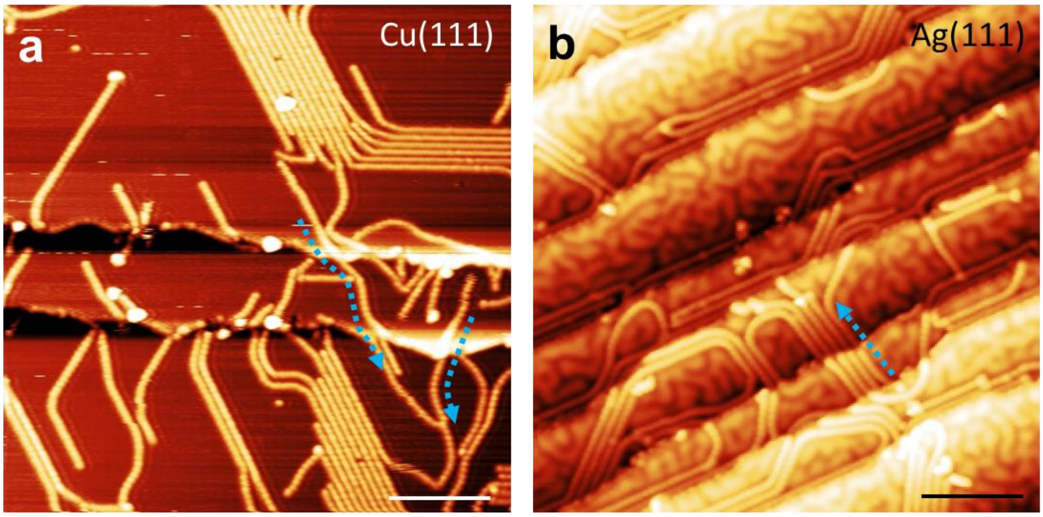

Supplementary Figure 4 | PPE nanowire arrays on metal surface. (a) The STM images of raft-like PPE nanowire arrays after annealing at 358 K on Cu(111) and on (b) Ag(111) substrates. Scale bars: 10 nm. After annealing at 358 K, elongated PPE nanowires can be observed crossing the Cu(111) steps by STM image. Blue arrows marked the nanowires across the atomic steps of substrates. We proposed that the PPE nanowire with passivated termini were mobile on Cu(111) (or Ag(111)) surface at 358 K and could cross the atomic steps to keep the orientations along the preferred direction, which was driven by the substrate-templating effect.

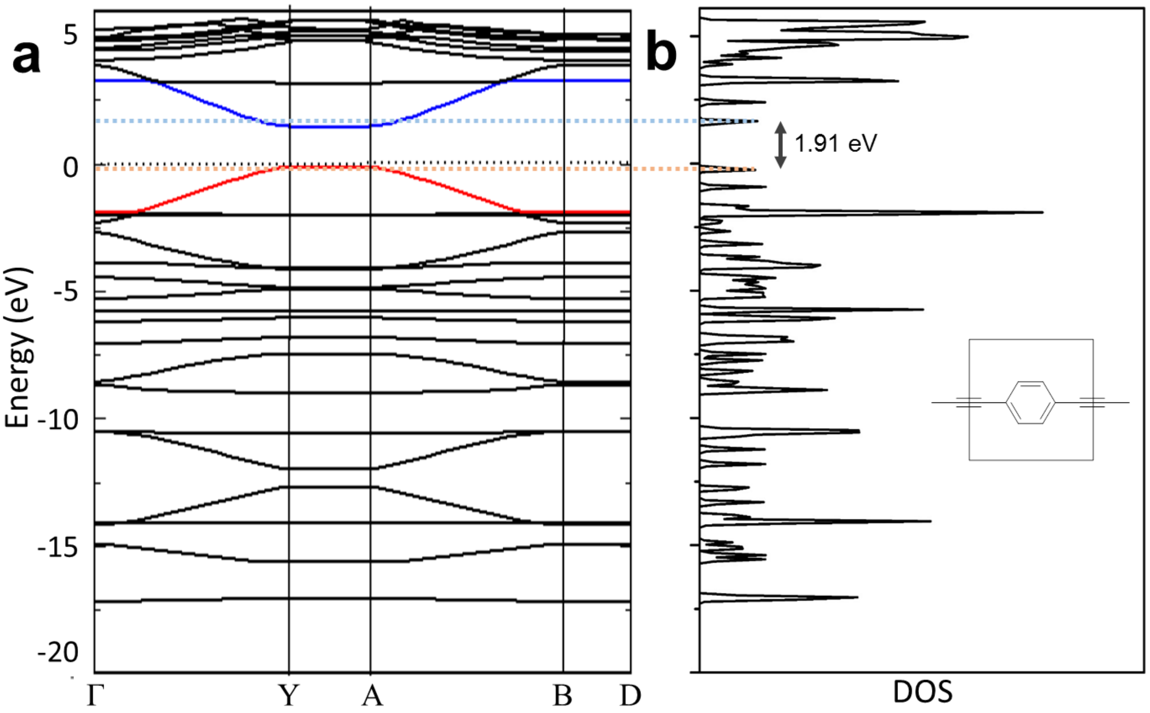

Supplementary Figure 5 | Band structure and the corresponding density of state (DOS) of gas-phase PPE nanowire. (a) The band structure of gas-phase PPE nanowire calculated by using DFT with the Perdew–Burke–Ernzerhof (PBE) exchange-correction functional. The significant dispersions were found along the axis of PPE nanowire [(Γ ➞ Y) and (A ➞ B)]. (b) The corresponding DOS exhibiting typical semiconductive feature with two resonance peaks near Fermi level. The energy splitting between the occupied state and the unoccupied state is ~ 1.91 eV.

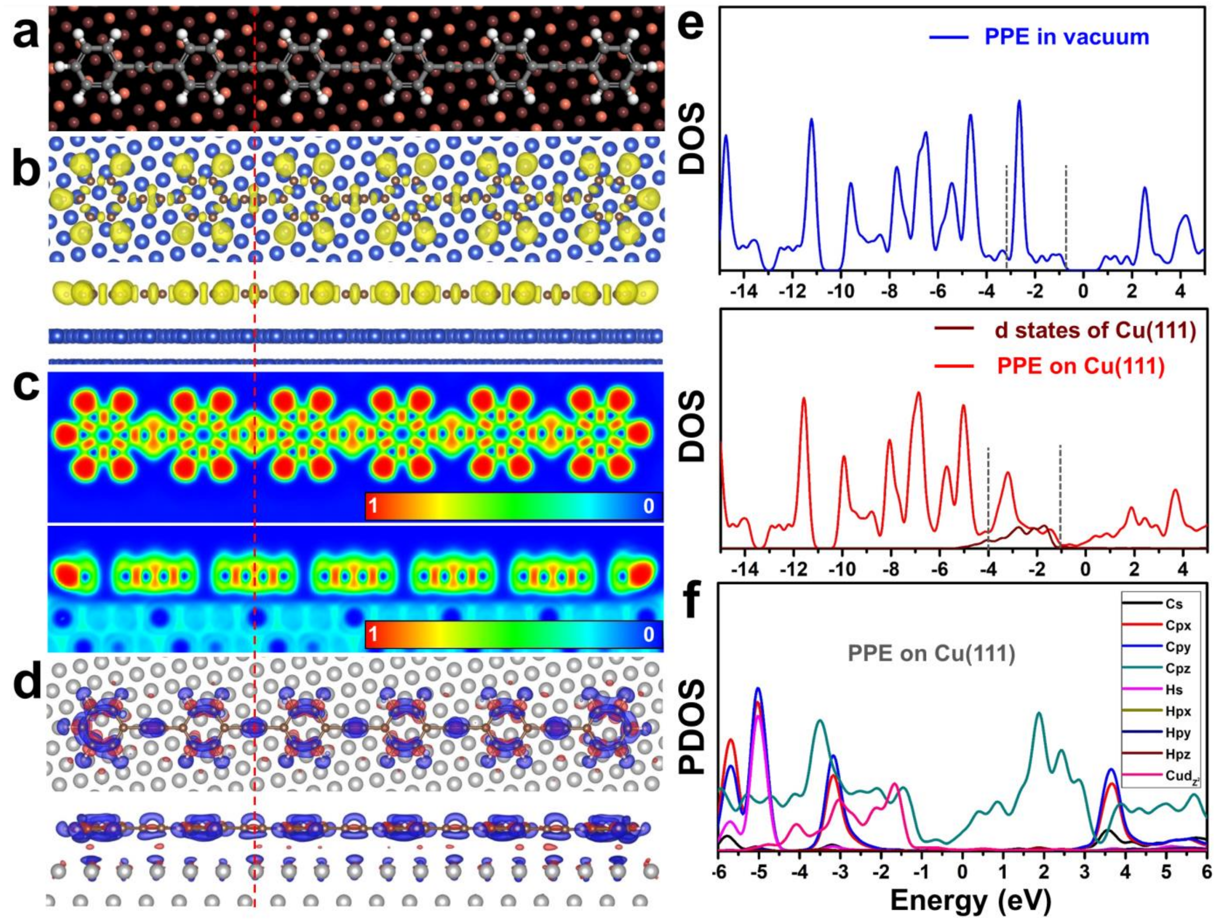

Supplementary Figure 6 | Interaction between PPE nanowire and the underlying Cu(111) surface. (a) DFT-optimized model of PPE nanowire on Cu(111). (b) Top-view and side-view images of electron localization function (ELF) localization domains (ELF value is 0.8) for PPE nanowire on Cu(111) surface[3,4] revealing significant larger electron localization domains around the C≡C bonds compared with the neighboring C-C single bonds. (c) Top and side views of ELF maps for PPE on Cu(111). It did not show any distinct chemical interaction between C≡C bonds and the underlying Cu(111) substrate. (d) The charge density difference (CDD) for PPE on Cu(111) ($\Delta\rho = \rho_{tot} - \rho_{slab} - \rho_{ads}$) with isodensity value of 0.0008 e/bohr^3. Red (blue) denotes positive (negative) regions. The red dash line indicates the location of carbon-carbon triple bond in PPE nanowire. A Bader charge analysis shows that only a small amount of charge transfer occurs from the Cu(111) to PPE nanowire of 0.03 e per unit (-Ar-C≡C-), suggesting the lack of electronic density sufficient for chemical bonding between PPE nanowire and Cu surface. Therefore, it is indicated that the PPE nanowires were physisorbed on Cu(111) substrate in this system. (e) The calculated density of states (DOS)

for the PPE nanowire in vacuum (upper panel) and on Cu(111) surface (lower panel) in a larger energy range than that in Figure 1f. The dark red line represents the d states of Cu(111). It can be observed that there is an overlap between the DOS of PPE on surface and the d states of Cu(111) surface in the energy range from -1.0 eV to -3.9 eV. (f) Projected density of states (PDOS) of PPE molecular wire on Cu(111) revealing the hybridization was mainly attributed to the interaction between p_z orbitals of PPE nanowire and d_{z^2} orbitals of Cu substrates.

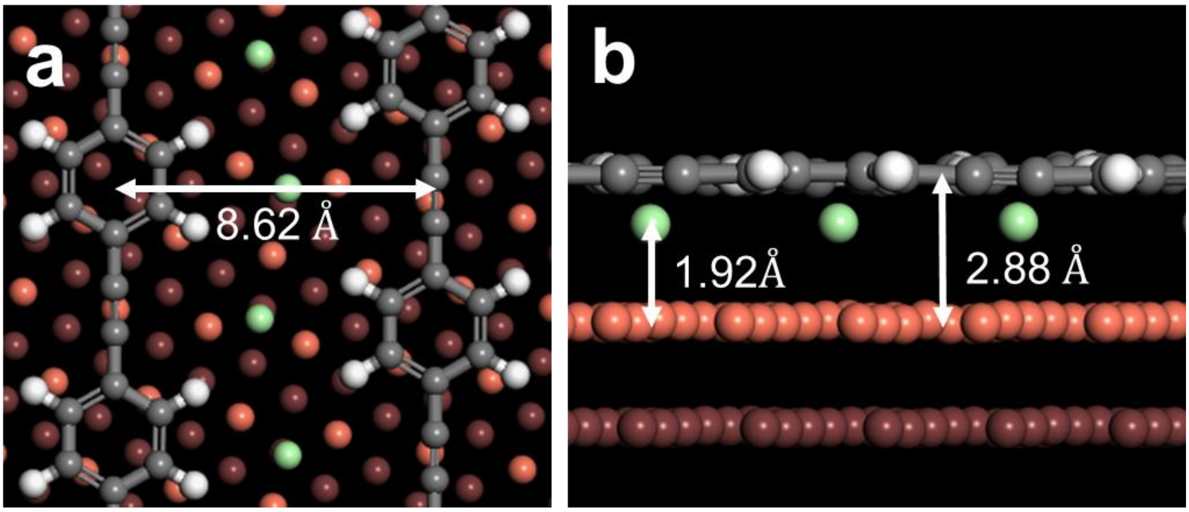

Supplementary Figure 7 | The DFT-optimized molecular structure of neighboring PPE nanowires adsorbed on Cu(111). (a) Top-view image. (b) Side-view image. DFT calculation results show that the Cl atoms located at hollow sites of Cu(111). The distance between neighboring PPE nanowires is ~ 8.62 Å, which agrees well with the experimental results (~ 9 Å). According to earlier publications[5,6], the Cl···H intermolecular bonds stabilized the neighboring PPE nanowire to form raft-like arrays.

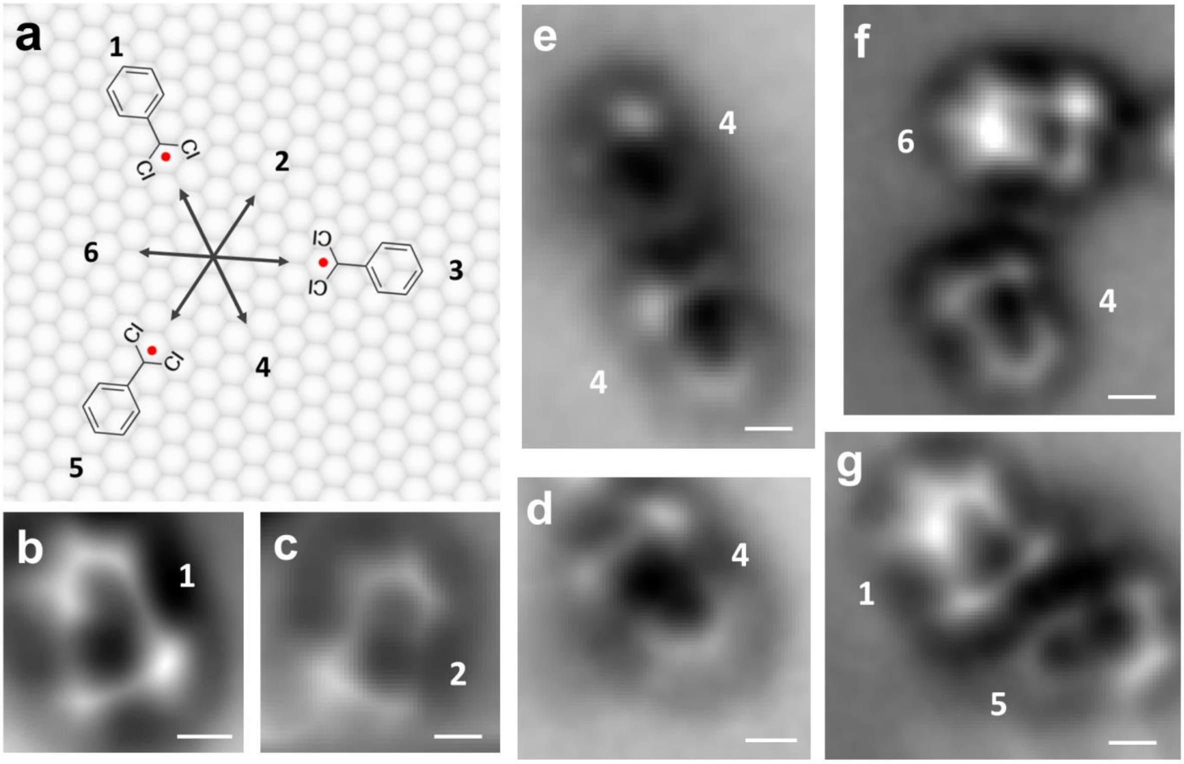

Supplementary Figure 8 | The identification of intermediates I by AFM measurements. (a) The diagram of optimal adsorption sites of intermediate I. For further clarifying the identification of the reaction intermediates by AFM image, a batch of AFM images of intermediate I (surface-stabilized benzyl radical) was acquired using different tips and on different samples. (b-d) Nc-AFM images of individual I on Cu(111). Scale bars: 300 pm. (e-g) Nc-AFM images of I dimers on Cu(111). Scale bars: 300 pm. Most AFM images of intermediate I showed similar feature of a tilted benzene ring and two neighboring protrusions. The AFM imaging distortion of reaction intermediates is mainly caused by tilting effect of CO on tip apex. According to the calculated model in Fig. 4j, the symmetry axis of surface-stabilized benzyl radical is along $[11\bar{2}]$ and the equivalent directions of Cu(111) surface. Thus there are six optimal adsorption sites, which were demonstrated in model diagram (Supplementary Fig. 8a). We found that the locations of all the intermediate I recorded in AFM images agreed with the adsorption sites predicted by DFT calculations.

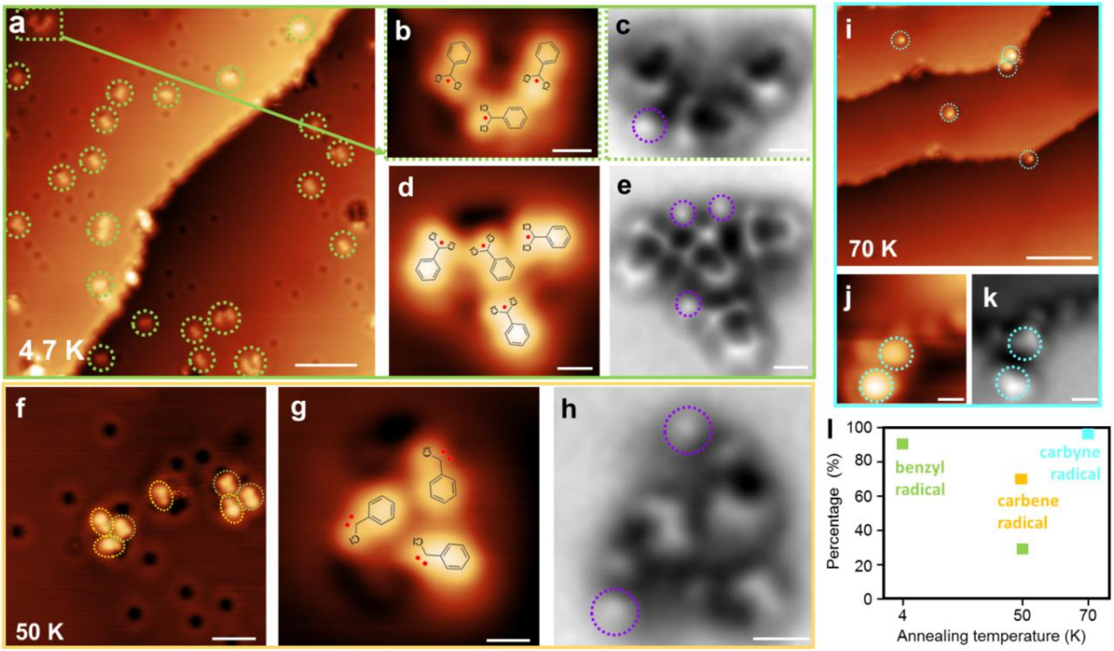

Supplementary Figure 9 | The overview of reaction intermediates observed after annealing at different temperature. (a) Large-scale STM image recorded after the TCMB deposited on Cu(111) at 4.7 K. (b, c) The close-up STM and AFM images of the magnified region of left top corner in Supplementary Figure 9a, revealed the molecules marked by green dashed circles could be identified to be benzyl radicals. (d, e) STM and AFM images of an island formed by four surface-stabilized benzyl radicals. We have counted the molecular species on Cu(111) formed at 4.7 K and nearly 90 % of them were benzyl radicals. (f) STM image recorded after annealing at ~ 50 K. A mixture of benzyl and carbene radicals was observed. Nearly 70% of the species after annealing at ~ 50 K were carbene radicals, while the left 30 % were benzyl radicals. (g, h) Close-up STM and AFM images of an island formed by three surface-stabilized carbene radicals. (i) Large-scale STM image recorded after elevated annealing at 70 K, even high than 70 K (in the range of 70 ~ 200 K). (j, k) Close-up STM and AFM images of surface-supported carbyne radicals on Cu(111). The coverage of molecule was decreased and only surface-supported carbyne radicals were observed (marked by blue dashed circles). (l) The statistic of intermediates after elevated annealing process. The necklace-like dots attached along the Cu steps in Supplementary Figure 9a, i were detached Cl atoms and the protrusions marked by purple dashed circles in Supplementary Figure 9c, e were adsorbed CO molecules. Scale bars: a, 5 nm; b, 6 Å; c-e, 5 Å; f, 2 nm; g, 5 Å; h, 4 Å; i, 10 nm; j, k, 4 Å.

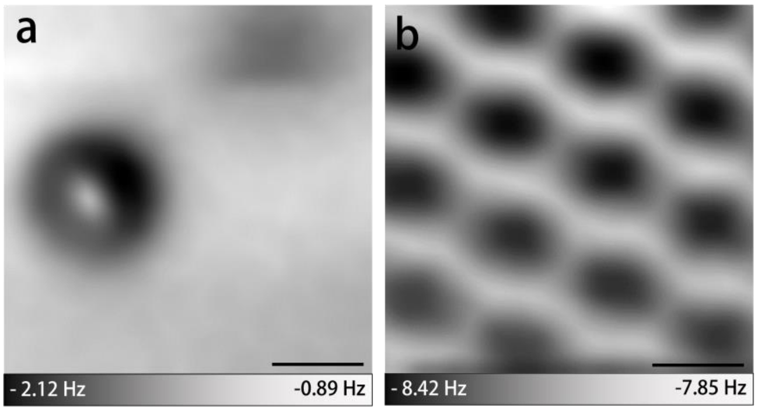

Supplementary Figure 10 | Measuring the height of radical III by nc-AFM. (a) The nc-AFM image of intermediate III at the height of tip set as 0 pm; scale bar: 500 pm. (b) The atomic resolved nc-AFM image of Cu(111) surface at the tip height of –7 Å. Scale bar: 200 pm. We applied nc-AFM to approximately measure the height of intermediate III. Firstly, we got the most clear nc-AFM image of intermediate III and set the height of tip as 0 pm (Supplementary Figure 10a). Then we moved the tip in horizontal direction to the nearest clean Cu(111) surface (on the same terrace) and approached to the Cu(111) surface until the most clear nc-AFM image was obtained (Supplementary Figure 10b) and recorded the tip height. The adsorption height of intermediate III can be approximately estimated as the approaching distance of tip. In this work, the height of III on Cu(111) is ~ 7 Å.

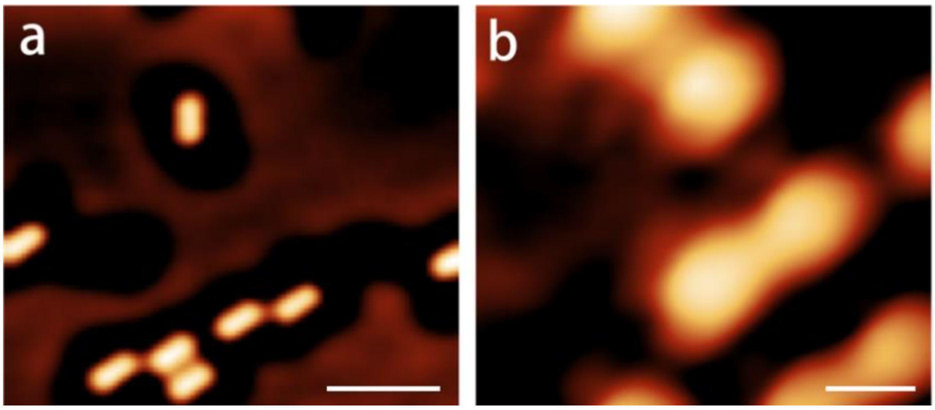

Supplementary Figure 11 | The STM images recorded after annealing the intermediate III to room temperature and re-cooled to 4.7 K (a) Large-scale and (b) close-up STM images of DPE molecules on Cu(111) showing similar feature with the directly-synthesized DPE molecules. The dark halo around DPE molecules in (a) can be assigned to the dissociated chlorine atoms. Scale bars: a, 3 nm; b, 5 Å.

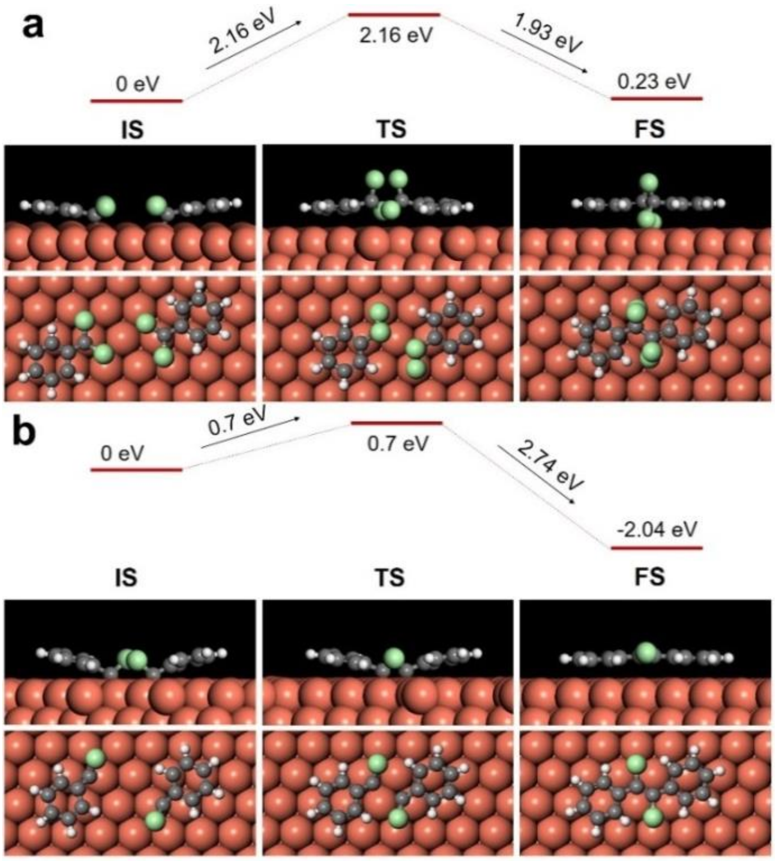

Supplementary Figure 12 | DFT calculation of different reaction pathways. The homo-coupling reaction barriers for (a) intermediate I and (b) intermediate II on Cu(111). Cu(111) surface were modeled by three layered slabs separated by at least 15 Å of vacuum (a_{Cu} = 3.59 Å). The bottom two layers were fixed. The oligomeric PPE adsorbed on a Cu (111) surface (13×7) and the structures were relaxed until the forces on all unconstrained atoms were ≤ – 0.05 eV/Å. The dechlorination of TCMB and the coupling of intermediate III were performed on a Cu(111) surface (5×5) and (4×7), respectively. The structures were relaxed until the forces on all unconstrained atoms were ≤ – 0.05 eV/Å. A 1×1×1 k-point sampling and a 400 eV kinetic energy cutoff were used. The nudged elastic band and Dimer methods were used for Transition-state calculations.

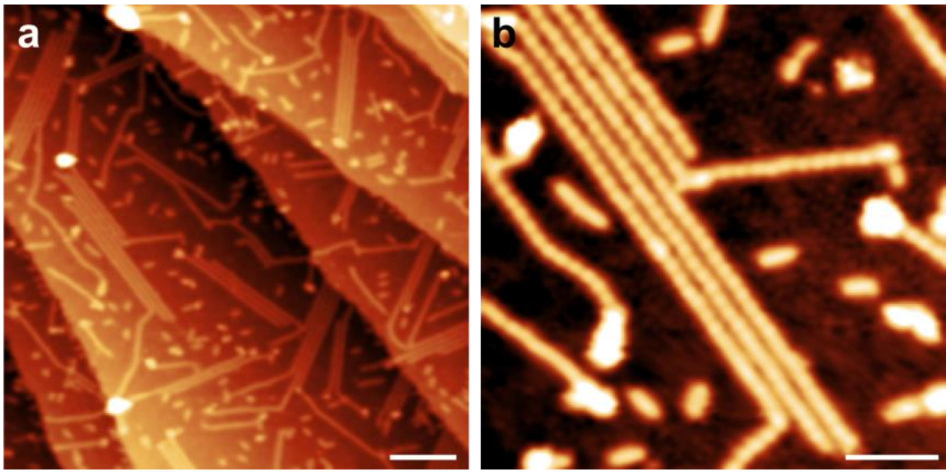

Supplementary Figure 13 | PPE nanowires synthesized on Ag(111) surface. (a) Large-scale STM image and the (b) close-up STM image of the PPE nanowire arrays synthesized on Ag(111). Scale bars: a, 10 nm; b, 4 nm.

Supplementary References:

1. Gutzler, R. *et al*. Surface mediated synthesis of 2D covalent organic frameworks: 1,3,5-tris(4-bromophenyl)benzene on graphite(001), Cu(111), and Ag(110). *Chem. Commun*. **45**, 4456-4458 (2009).
2. Schuler, B. *et al.* Unraveling the Molecular Structures of Asphaltenes by Atomic Force Microscopy. *J. Am. Chem. Soc.* **137**, 9870-9876 (2015).
3. Silvi, B. & Savin, A. Classification of chemical bonds based on topological analysis of electron localization functions. *Nature* **371**, 683-686 (1994).
4. Santos, J. C. Polo, V. Andrés, J. An electron localization function study of the trimerization of acetylene: Reaction mechanism and development of aromaticity. *Chem. Phys. Lett.* **406**, 393-397 (2005).
5. Wang, W. *et al.* Single-Molecule Resolution of an Organometallic Intermediate in a Surface-Supported Ullmann Coupling Reaction. *J. Am. Chem. Soc*. **133**, 13264-13267 (2011).
6. Park, J. *et al.* Interchain Interactions Mediated by Br Adsorbates in Arrays of Metal-Organic Hybrid Chains on Ag(111). *J. Phys. Chem. C*. **115**, 14834-14838 (2011).
